# Hsa-miR-223-3p participates in the process of anthracycline-induced cardiomyocyte damage by regulating NFIA gene

**DOI:** 10.1515/med-2023-0754

**Published:** 2023-07-25

**Authors:** Xiao Han, Kun Liu, Fumin Gao, Mingjun Yang, Fei Wang

**Affiliations:** Department of Cardiothoracic Surgery, Affiliated Hospital of Nantong University, 20 Xisi Road, Nantong, Jiangsu, 226001, China; Department of Cardiothoracic Surgery, Affiliated Hospital of Nantong University, Nantong, Jiangsu, 226001, China

**Keywords:** miR-223-3p, NFIA, cardiomyocyte damage, anthracyclines

## Abstract

Irreversible cardiomyopathy was caused by the therapeutic of anthracyclines in the chemotherapy of cancers. The cell apoptosis and autophagy were induced by anthracyclines in AC16 cells. MiR-223-3p ascends in anthracycline-treated AC16, but the expression of nuclear factor I-A (NFIA) was specifically down-regulated. However, the underlying molecular mechanism between NFIA and miR-223-3p is unclear now in AC16 cells. In our research, NFIA expression was dampened in AC16 cells by miR-223-3p mimics. Additionally, miR-223-3p knockdown hindered the apoptosis and autophagy in anthracycline-treated AC16. Furthermore, NFIA was predicted and verified as a miR-223-3p’s downstream target and rescued the functions of miR-223-3p. These findings illustrated that miR-223-3p advances anthracycline-stimulated cardiomyocyte damage progression by targeting NFIA, implying the promising therapeutic function of miR-223-3p on cardiomyocyte damage in cancer patients.

## 1 Introduction

Cancer remains a leading cause of death worldwide, and anthracyclines are widely used in the chemotherapy of many solid and hematological cancers [1]. But the dose-dependent relationship between anthracyclines and congestive heart failure caused by irreversible cardiomyopathy hinders their therapeutic potential [2]. As key regulators of cellular progression associated with drug resistance [3], microRNAs (miRNAs) are demonstrated to play suppressor roles in different oncogenic and tumor types [4,5]. This phenomenon is related to the regulation of a variety of complex pathways, including the loss of drug accumulation ability, the protective mechanism of autophagy, the reduction of DNA damage, and the inhibition of downstream signals from turning into apoptosis events [3].

miRNAs are generally composed of 18–25 nucleotides and are highly conserved noncoding RNAs, which modulate the expression of multiple genes [6]. In addition to their role in controlling the mechanisms of chemo-resistance, miRNAs also have shown that changes in their levels are related to multiple cardiovascular diseases and heart tissue damage [7,8]. We predicted genes related to cardiomyocyte damage and found that most of them are related to miR-223-3p, and then found that many reports had revealed miR-223-3p was relative to cell apoptosis promotion [9,10] and cell proliferation inhibition [11]. It was reported that the reduction of miR-223-3p lessened the occurrence of ischemic arrhythmias [12]. In H9c2 cells, deletion of miR-223-3p significantly lessened cardiomyocyte damage induced by hypoxia, including improving cell viability, which indicates a new mechanism for miR-223-3p to prevent the apoptosis and oxidative stress through modulating KLF15 expression in cardiomyocyte [13]. However, the molecular mechanisms that miR-223-3p regulates the evolvement of anthracycline-induced cardiomyocyte damage have not been fully clarified.

In our research, we predicted that miR-223-3p was interrelated to nuclear factor I-A (NFIA) by bioinformatics in AC16 cells. Research studies make known that miR-212-3p induces apoptosis of breast cancer cells by targeting NFIA [14] and miRNA-302b induces glioma cell apoptosis by regulating NFIA/insulin-like growth factor-binding protein 2 signaling pathway [15]. So, our research focus was the pathological effect of miR-223-3p in AC16 cells treated with anthracycline, as well as the possible molecular mechanisms of both NFIA and miR-223-3p.

All data demonstrated that miR-223-3p increased apoptosis and autophagy through binding with NFIA, which was a new molecular regulation mechanism in the process of anthracycline-induced cardiomyocyte damage.

## 2 Methods and materials

### 2.1 Cell cultivation and processing

Human cardiomyocyte-like cells (AC16) acquired from the American Type Cell Culture (Rockville, MD, USA) were cultivated in DMEM (Bio-Channel, Nanjing, China) supplementary with 10% fetal bovine serum (OriCell, Suzhou, China) at 37°C, 5% CO_2_. Cells were processed with 1 μM epirubicin and 1 μM pirarubicin for 24 h.

### 2.2 Gene transfection

Human miR-223-3p mimic, the human miR-223-3p inhibitor, NFIA siRNA (siNFIA), and its negative control siRNA (siNC) (RiboBio, Guangzhou, China) were transfected with cells. For transfection, inoculated AC16 in six-hole plates and transfected with 100 nM miR-223-3p mimic, miR-223-3p inhibitor, or siNFIA as well as their corresponding negative control by Lipofectamine 2000 (GLPBIO, Montclair, CA, USA) following the protocol.

### 2.3 Cell apoptosis

Estimated cell apoptosis by flow cytometry. Briefly, after cell collection, suspended with binding buffer (100 μL), and stained in the dark by propidium iodide and FITC Annexin V. After 10 min, the binding buffer (400 μL) was added to each tube. Subsequently, tested cell apoptosis by flow cytometry (Agilent Technologies, USA), and analyzed the data by FlowJo.

### 2.4 TUNEL assay

We detected the apoptosis using a TUNEL assay. The assay was carried out with a commercial Cell Death Detection kit (Roche Molecular Diagnostics) following the manufacturer’s kit’s protocols.

### 2.5 Transmission electron microscopy (TEM)

Under TEM, fixed the samples for 30 min in 2.5% glutaraldehyde, and treated at 20°C for 1 h with 1% buffered osmium tetroxide. Subsequently, the increasing ethanol gradient was used to dehydrate the samples followed by embedding in epoxy resin. Finally, observed and recorded the samples under a TEM (HT7800; HITACHI, Tokyo, Japan).

### 2.6 Western blotting

Extracted and calculated total proteins using a BCA assay kit (TIANGEN, Beijing, China). First, transferred the samples onto PVDF (Millipore, 0.45 μm) membranes after separation in 10% SDS-PAGE. The primary antibodies (Abcam, Cambridge, UK) were anti-NFIA (ab228897, 1:800), anti LC3-Ⅱ (ab48394, 1:800), anti-cleaved Caspase-3 (ab32042, 1:1,000), anti-Caspase-3 (ab13847, 1:500), anti-Caspase-9 (ab32539, 1:1,000), anti-cleaved Caspase-9 (ab2324, 1:1,000), anti-p62 (ab56416, 1:1,000), and anti-β-actin (ab8226, 1:800). Immunoreactive membranes were observed using ECL detection reagent (Millipore). The density of the bands was quantified by ImageQuant 1D software (GE Healthcare, PA, USA). The internal control was β-actin.

### 2.7 Quantitative real-time PCR (qRT-PCR) assay

Extracted total RNA in the Trizol reagent (Beyotime, Shanghai, China) following the protocols. The machine for qPCR was from Life Technologies Holdings Pte Ltd, Shanghai, China. The reactions were implemented with SYBR^®^ Green Master Mix (Takara, Japan). Table 1 shows the primers for RT-qPCR. Normalized the relative expression levels of NFIA and miR-223-3p, respectively, to U6 and GAPDH.

**Table 1:** Primer sequences used for qRT-PCR

Genes		Primer sequences (5′–3′)
hsa-miR-223-3p	Forward	GGTAACAGTCTCCAGTCA
	Reverse	GCAATTGCACTGGATACG
NFIA	Forward	TGGCCAAGTTACGGAAAGATG
	Reverse	GCGCTCGCCATCAGTACT
U6	Forward	GCTTCGGCAGCACATATACTAAAAT
	Reverse	CGCTTCACGAATTTGCGTGTCAT
GAPDH	Forward	GAAGGTGAAGGTCGGAGTC
	Reverse	GAAGATGGTGATGGGATTTC

### 2.8 Luciferase assay

We predicted the binding sites of miR-233-3p by utilizing bioinformatics. WT and mutated (MUT) NFIA 3′-UTR sequences were chemically synthesized (Sangon, Shanghai, China) according to the sequence of miR-223-3p. Besides, the luciferase activity was detected according to the luciferase assay system (Promega, USA).

### 2.9 Statistics analysis

Comparisons were measured through unpaired/paired Student’s *t*-test, Mann–Whitney test, one-way ANOVA, and Bonferroni’s *post hoc* test. By SPSS 18.0 (SPSS Inc.), data were shown as the mean  ±  standard deviation. The differences were deemed statistically significant at *P*  <  0.05.

## 3 Results

### 3.1 Anthracyclines induce the apoptosis and autophagy of AC16 cell

**Figure 1:**
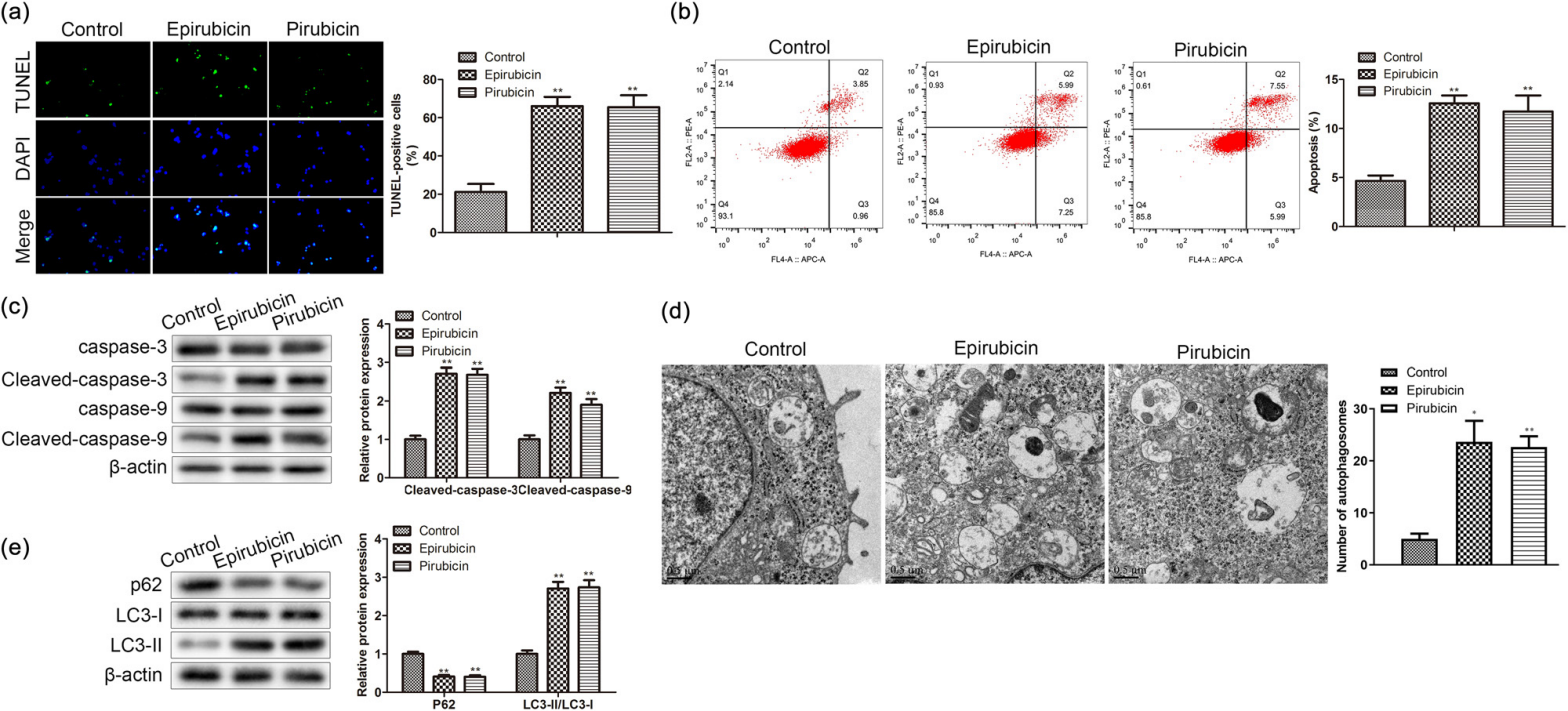
Anthracyclines induce AC16 cell apoptosis and autophagy. AC16 were processed with 1 μM epirubicin and 1 μM pirarubicin for 24 h. (a) and (b) Apoptosis was measured by TUNEL and flow cytometry in AC16 cells. (c) Levels of cleaved caspase-3 and -9 were assessed by western blot analysis in AC16 cells. (d) Autophagosomes were tested by TEM. (e) Expressions of p62, LC3-I, and LC3-II in AC16 cells. **P < 0.01 vs control. *n* = 3 per group.

Epirubicin and pirarubicin exert their anti-tumor effects through complexes with DNA, leading to DNA damage and interfering with the synthesis of DNA, RNA, and proteins [16]. AC16 cells were stimulated with 1 μM epirubicin and 1 μM pirarubicin for 1 day. TUNEL-positive cell quantity measured in AC16 cells was enhanced by epirubicin and pirarubicin (Figure 1(a)). Flow cytometry assay elucidated an elevating apoptosis rate triggered by epirubicin and pirarubicin in AC16 cells (Figure 1(b)). Then western blot was applied to evaluate apoptosis associated proteins’ content. We discovered that pro-apoptosis proteins’ level (Cleaved caspase-3 and -9) was elevated by epirubicin and pirarubicin in AC16 cells (Figure 1(c)). Next, TEM showed an increase in the number of autophagosomes with typical bilayer morphology in AC16 cells treated with epirubicin and pirarubicin (Figure 1(d)). Furthermore, the level of signaling adaptor p62 was decreased, yet LC3-II level was elevated by epirubicin and pirarubicin in AC16 cells (Figure 1(e)). All the results showed that anthracyclines promote apoptosis and autophagy in AC16 cells.

### 3.2 Anthracyclines promote miR-223-3p expression and cell damage

**Figure 2:**
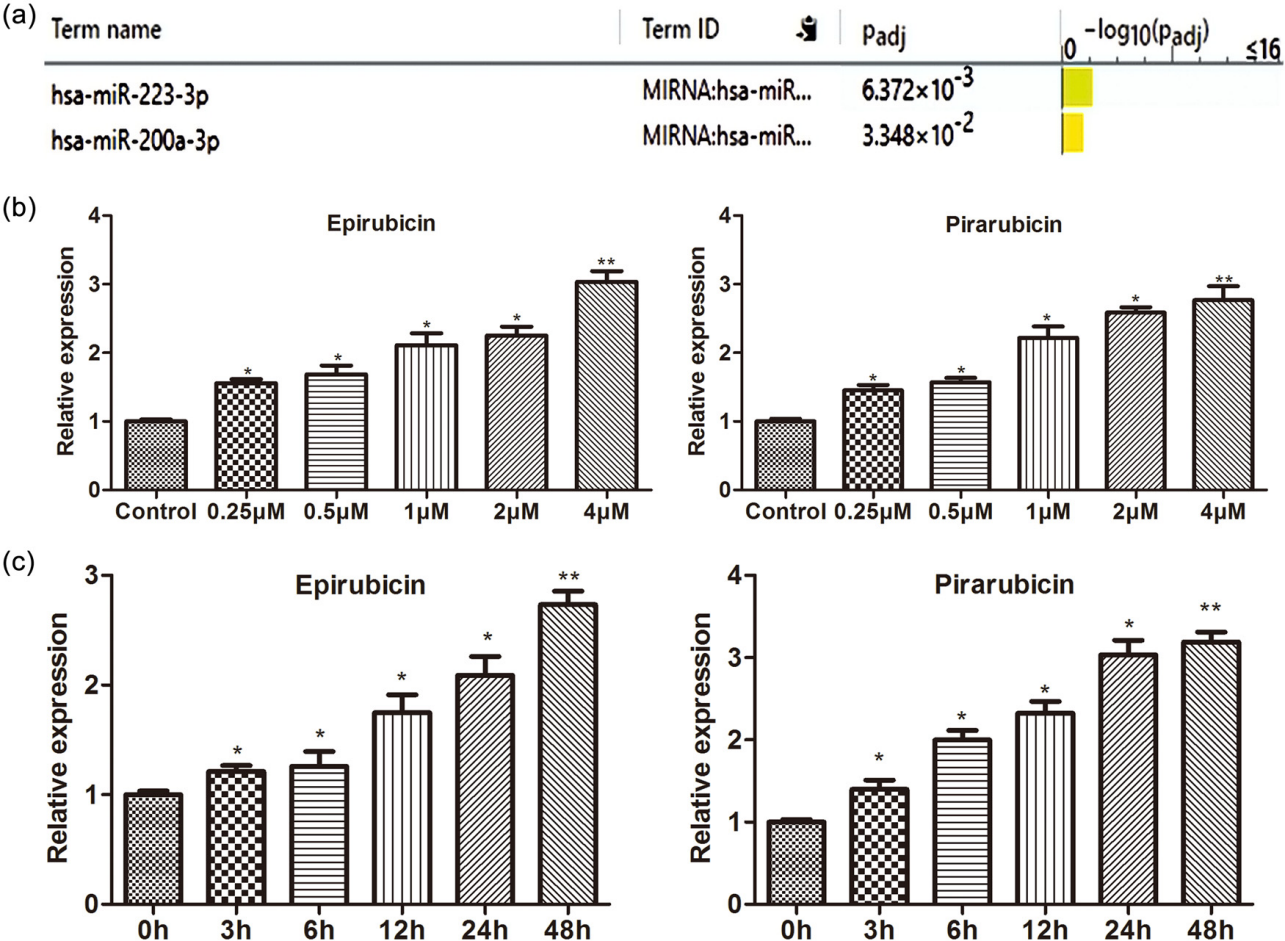
Anthracyclines promote miR-223-3p level. (a) Genecards were used to search the genes related to cardiomyocyte damage and then found most of them are related to miR-223-3p. (b) and (c) miR-223-3p levels were assessed by qRT-PCR in AC16 cells treated with different concentrations of epirubicin and pirarubicin or different time points of epirubicin and pirarubicin. *P < 0.05, **P < 0.01 vs 0 h; *P < 0.05, **P < 0.01 vs control. *n* = 3 per group.

We found miRNAs related to myocardial cell injury through Genecards (https://www.genecards.org/) and g: Profiler (https://biit.cs.ut.ee/gprofiler/) enrichment analysis, with miR-223-3p having a higher correlation with myocardial cell injury (Figure 2(a)). miR-223-3p was reported to induce cell damage, especially in cardiomyocytes [12]. Different concentrations of epirubicin and pirarubicin were used to treat AC16 cells, and the qPCR results implied that miR-223-3p expression was ascended through epirubicin or pirarubicin in the dose-dependent manner (Figure 2(b)). Then AC16 cells were treated with epirubicin or pirarubicin for different concentrations or hours, we discovered a constant increasing miR-223-3p level with the prolongation of epirubicin or pirarubicin treatment time (Figure 2(c)). Above data confirmed that miR-223-3p content was positively related to anthracyclines treatment.

### 3.3 miR-223-3p down-regulation affects the apoptosis of AC16 cell

**Figure 3:**
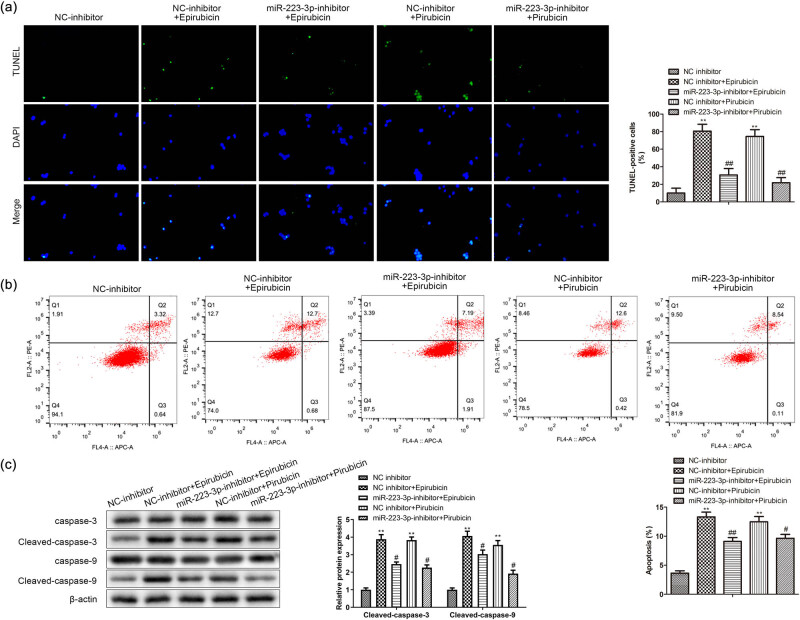
miR-223-3p knockdown affects AC16 apoptosis. miR-223-3p inhibitor and NC inhibitor were transfected into AC16 cells treated with epirubicin or pirarubicin. (a) and (b) Apoptosis was measured by TUNEL and flow cytometry in AC16 cells. (c) Levels of cleaved caspase-3 and -9 in AC16 cells were assessed by western blot analysis. **P < 0.01 vs NC-inhibitor; ^#^ *P* < 0.05, ^##^ *P* < 0.01 vs NC-inhibitor + epirubicin, NC-inhibitor + pirarubicin. *n* = 3 per group.

To probe the effects of miR-223-3p, AC16 cells treated with epirubicin or pirarubicin were transfected with miR-223-3p inhibitor and NC inhibitor, respectively. Compared to the NC inhibitor group, the TUNEL-positive AC16 cells were reduced in miR-223-3p inhibitor group, signifying that apoptosis degree was cut down by miR-223-3p reduction in the AC16 stimulated with epirubicin or pirarubicin (Figure 3(a)). The flow cytometry elucidated that miR-223-3p knockdown suppressed cellular apoptosis in the AC16 cells treated with epirubicin or pirarubicin (Figure 3(b)). In addition, expressions of pro-apoptosis proteins (cleaved caspase-3 and -9) were discovered to be debased by a miR-223-3p inhibitor (Figure 3(c)). Above results showed that the apoptosis degree up-regulated by epirubicin or pirarubicin was inhibited by miR-223-3p inhibitor in AC16.

### 3.4 Down-regulation of miR-223-3p affects AC16 cell autophagy

**Figure 4:**
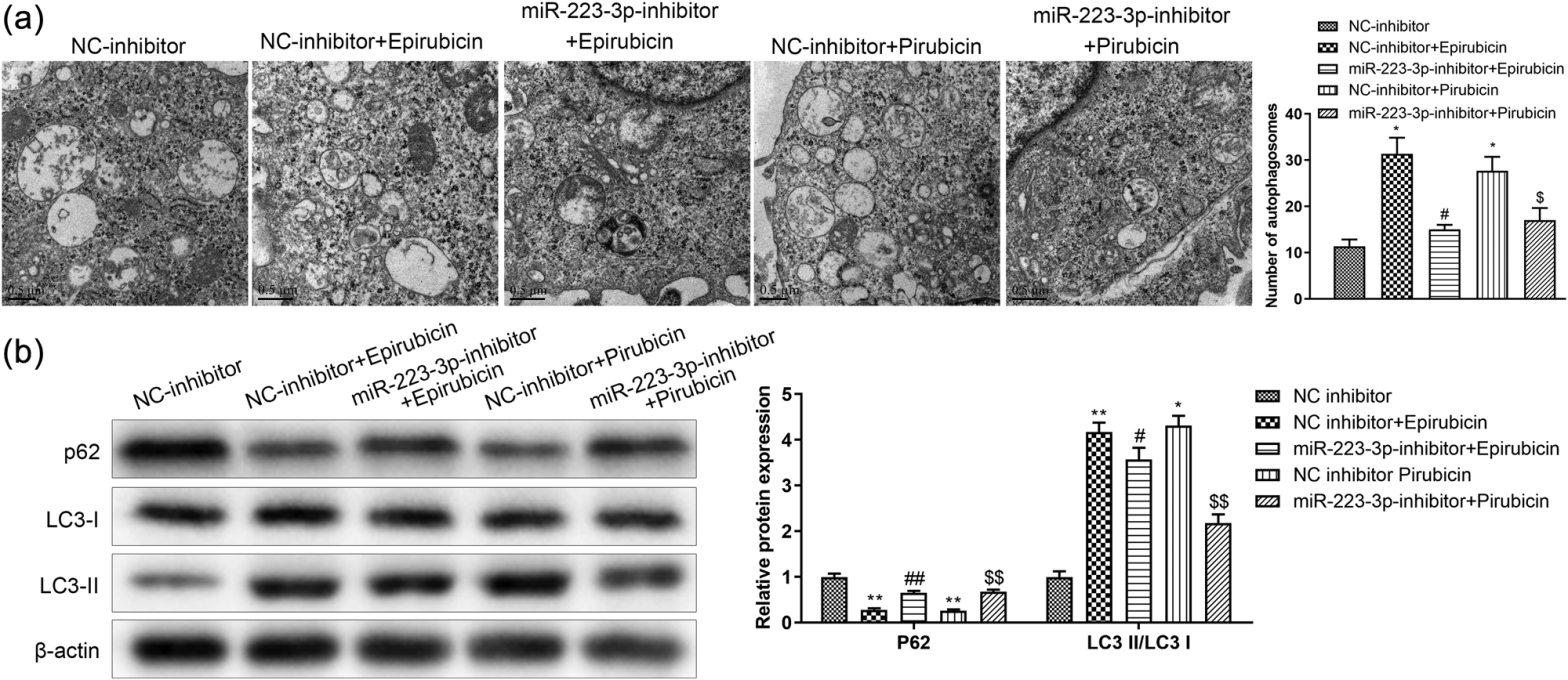
miR-223-3p knockdown affects AC16 autophagy. miR-223-3p inhibitor and NC inhibitor were transfected into AC16 cells treated with epirubicin or pirarubicin. (a) Autophagosomes were detected by TEM. (b) Expressions of p62, LC3-I, and LC3-II were measured by western blot analysis in AC16 cells. *P < 0.05, **P < 0.01 vs NC-inhibitor; ^#^ *P* < 0.05, ^##^ *P* < 0.01 vs NC-inhibitor + epirubicin, NC-inhibitor + pirarubicin. *n* = 3 per group.

To inspect the role of miR-223-3p in the autophagy of AC16 cells, electron microscopy was used to detect the autophagosome. Results suggested that the increased number of autophagosomes was reduced by knockdown of miR-223-3p in AC16 stimulated with epirubicin or pirarubicin (Figure 4(a)). In AC16 cells treated with epirubicin or pirarubicin, the results of western blot showed that miR-223-3p inhibitor promoted LC3-Ⅱ protein level, but abated p62 protein level (Figure 4(b)). All these data illustrated that low miR-223-3p level reduces the level of autophagy in AC16 cells.

### 3.5 Analyzed and verified the relevance between NFIA and miR-223-3p

**Figure 5:**
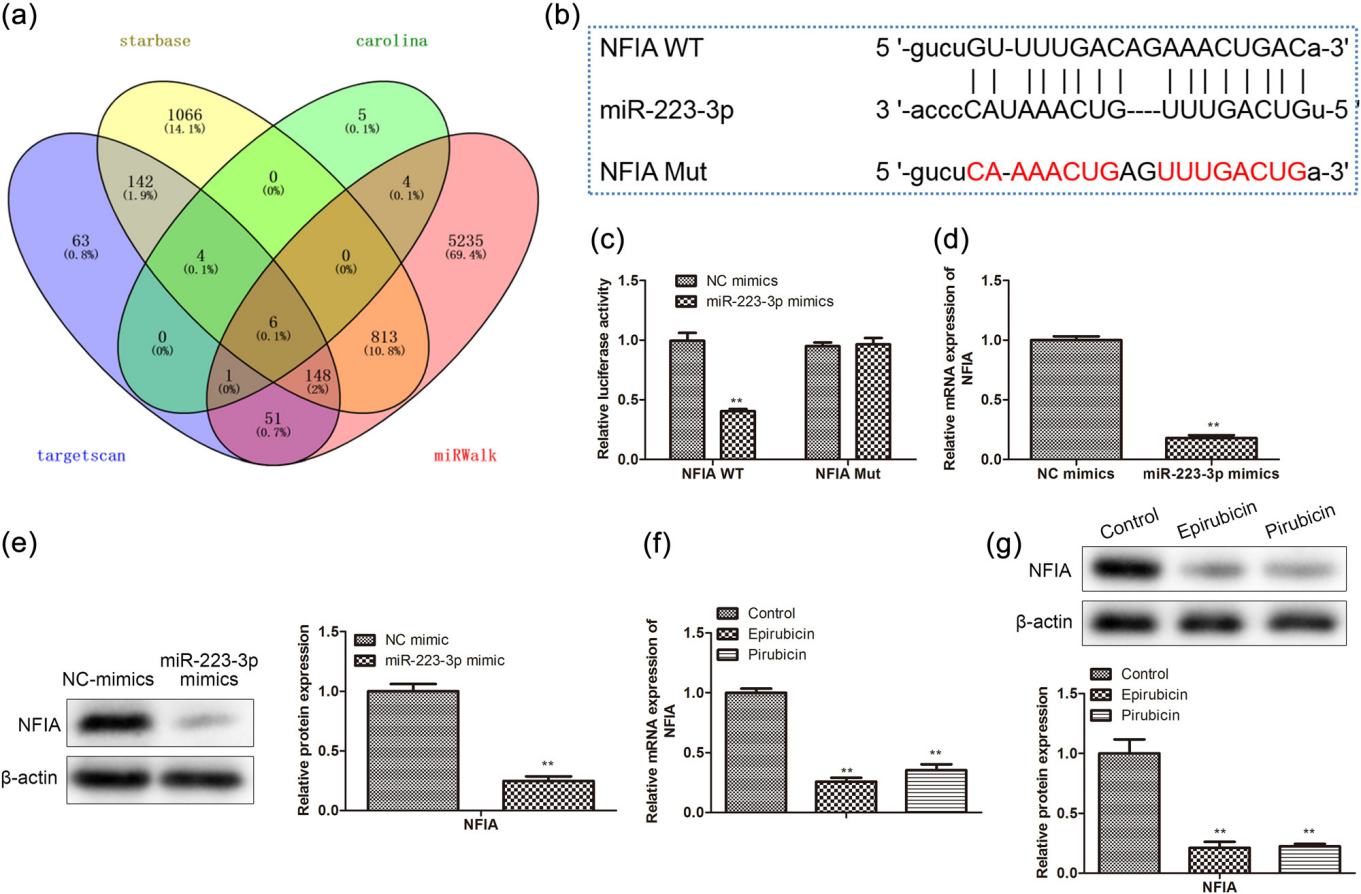
Analyze and verify the relevance between NFIA and miR-223-3p. (a) We used bioinformatics prediction websites to predict the relationship and (b) the binding site of both miR-223-3p and NFIA. (c) Luciferase reporter assay was performed after 48 h transfection in AC16 with a luciferase reporter plasmid containing WT or mutant form of NFIA 3′-UTR along with NC mimics or miR-223-3p mimics. NC mimics or miR-223-3p mimics were transfected in AC16 cells, and NFIA expression was analyzed by (d) qRT-PCR and (e) western blot. The protein level of NFIA treated with epirubicin or pirarubicin was tested by (f) qRT-PCR and (g) western blot. **P < 0.01 vs NC mimics; **P < 0.01 vs NC-inhibitor + si-NC; ^#^ *P* < 0.05, ^##^ *P* < 0.01 vs miR-223-3p inhibitor + si-NC; ^$^ *P* < 0.05, ^$$^ *P* < 0.01 vs miR-223-3p-inhibitor + Si-NC. *n* = 3 per group.

It was proved that the NFIA transcription factor participates in inhibiting cell apoptosis [17]. We used bioinformatics prediction websites (starbase, Carolina, targetscan, and miRWalk) to predict the relationship and the binding site between NFIA and miR-223-3p (Figure 5(a) and (b)). Then, luciferase reporter analysis was carried out, showing that luciferase activity of the 3′-UTR region of NFIA dramatically reduced in AC16 co-transfected with NFIA-WT and miR-223-3p mimics. However, luciferase activity of NFIA-Mut showed no significant difference between the NC group and miR-223-3p mimics group (Figure 5(c)). Meanwhile, qRT-PCR and western bolt illustrated that NFIA level was specifically depressed by miR-223-3p mimics (Figure 5(d) and (e)). Then it was found that the NFIA level was reduced by anthracyclines (Figure 5(f) and (g)). In summary, the above data illustrated that miR-223-3p binds with NFIA mRNA and negatively regulates NFIA expression.

### 3.6 Rescue experiments verified NFIA/miR-223-3p’s role in anthracycline-treated AC16

**Figure 6:**
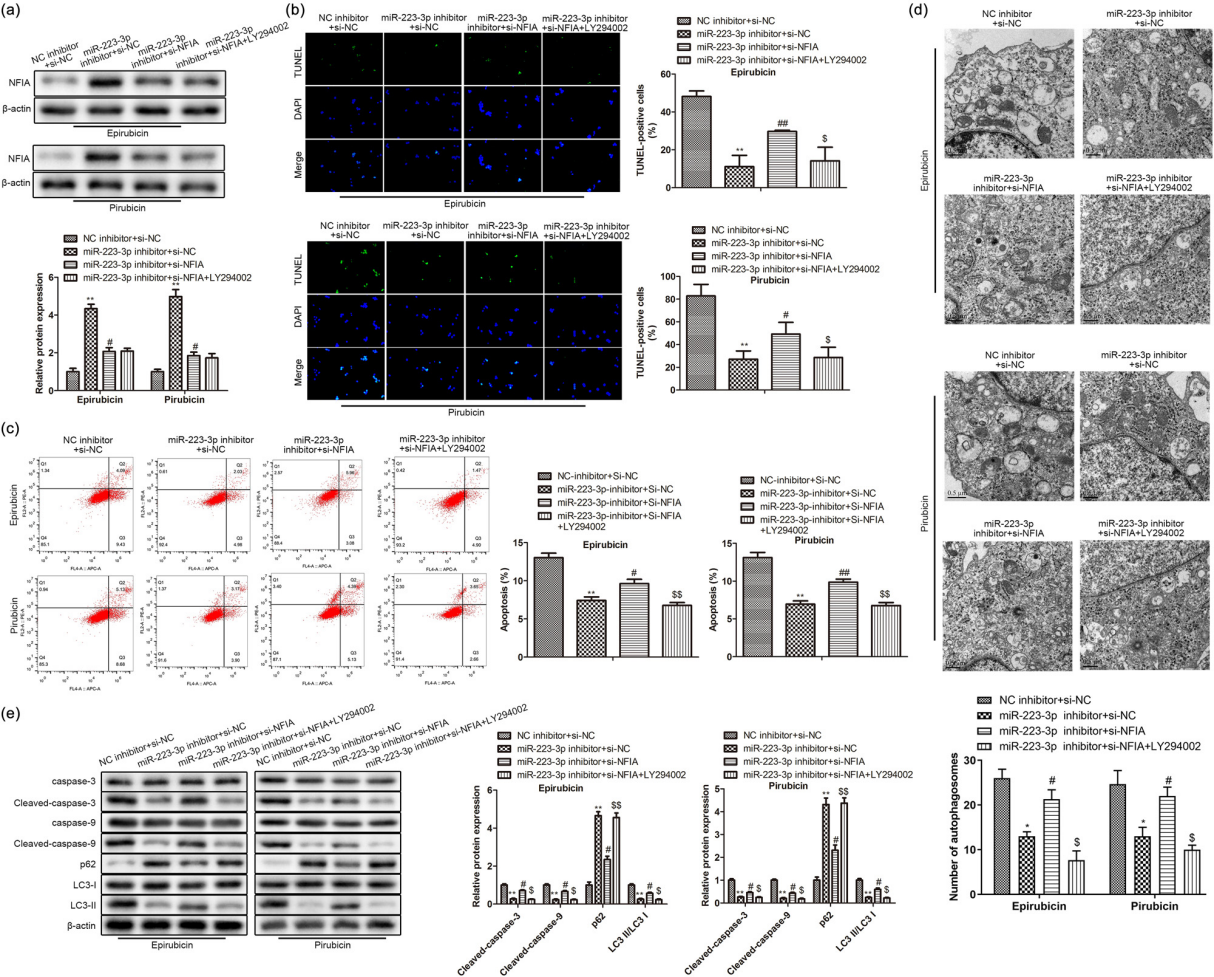
Rescue experiments verify the effects of NFIA/miR-223-3p in anthracycline-treated AC16. miR-223-3p inhibitor/NC inhibitor and si-NFIA/si-NC were co-transfected into anthracycline-induced AC16 cells processed with or without LY294002, respectively. (a) NFIA expression was detected by western blot. (b) and (c) Apoptosis of AC16 cells was detected by TUNEL and flow cytometry. (d) Autophagosomes were detected by TEM. (e) Expression levels of p62, LC3-I, LC3-II, cleaved caspase-3 and -9. **P < 0.01 vs normal. *n* = 3 per group.

To test the functions of the NFIA/miR-223-3p axis, miR-223-3p inhibitor/NC inhibitor and si-NFIA/si-NC were co-transfected into AC16 cells treated with epirubicin or pirarubicin, respectively. NFIA up-regulation by miR-223-3p inhibitor was dampened by si-NFIA in the AC16 cells processed with epirubicin or pirarubicin, and the expression of NFIA was not affected by autophagy inhibitor LY294002 (Figure 6(a)). The reduction of the TUNEL-positive AC16 cells was improved by si-NFIA in miR-223-3p inhibitor group, and it was inhibited by LY294002 in AC16 cells treated with epirubicin or pirarubicin. It showed that the decreasing of apoptosis degree by miR-223-3p inhibitor was reversed by si-NFIA in AC16 cells treated with epirubicin or pirarubicin, and the autophagy inhibitor LY294002 inhibited the up-regulation of apoptosis by si-NFIA (Figure 6(b)). The flow cytometry assay elucidated that si-NFIA inverted the inhibition on apoptosis by miR-223-3p inhibitor in the AC16 cells treated with epirubicin or pirarubicin, and the autophagy inhibitor LY294002 inhibited the up-regulation of cell apoptosis by si-NFIA (Figure 6(c)). Next, si-NFIA reversed the decrease of autophagosomes by miR-223-3p inhibitor in the AC16 treated with epirubicin or pirarubicin, and the autophagy inhibitor LY294002 inhibited the increase of autophagosomes by si-NFIA (Figure 6(d)). Furthermore, miR-223-3p silencing stimulated the decline of pro-apoptosis factor and LC3-Ⅱ was partly reversed by si-NFIA by western bolt analysis; meanwhile, the increase was rescued by LY294002. The descent by miR-223-3p inhibitor in p62 expression was partly reversed by si-NFIA; meanwhile, the reduced effect on p62 was rescued by LY294002 (Figure 6(e)). All the results showed that si-NIFA reversed miR-223-3p inhibitor effects of anti-apoptosis and anti-autophagy in anthracycline-treated AC16, indicating that miR-223-3p monitored the process of apoptosis and autophagy by targeting NFIA in the anthracycline-treated AC16.

## 4 Discussion

The anthracycline drugs epirubicin and pirarubicin are considered a double-edged sword in clinical treatment. On the positive side, anthracyclines exert a capital role in the therapy of neoplastic disorder; on the negative side, chronic use of anthracyclines spawns congestive heart failure and cardiomyopathy, which are often difficult to cure with common drugs [18]. This study found that epirubicin or pirarubicin increased autophagy and apoptosis of AC16 cells. According to reports, the expression of miRNA is affected by drugs, and their levels are associated with various cardiovascular diseases and heart tissue damage, indicating that miRNA has the potential as a biomarker and diagnostic tool [8]. This study found that the expression level of miR-223-3p was significantly higher in AC16 cells treated with epirubicin and pirarubicin.

Many miRNAs are involved in the progression of cardiomyocyte damage. For instance, it is reported that the knockdown of miR-132 improves heart failure and cardiac hypertrophy in mice [19]. Also, miR-34a was found to spawn in the aging heart, and miR-34a knockdown reduces the death of age-associated cardiomyocytes *in vivo* [20]. Meanwhile, miR-223-3p prevents oxidative stress and apoptosis via regulating KLF15 in H9c2 cells [13]. In this study, we predicted genes related to cardiomyocyte damage and found that most of them are related to miR-223-3p, which was up-regulated by epirubicin or pirarubicin in AC16 cells. Furthermore, miR-223-3p reduction hindered the apoptosis and autophagy in AC16 cells treated with epirubicin or pirarubicin. Those results revealed that miR-223-3p is a crux for the restraint of apoptosis and autophagy in anthracycline-treated AC16.

NFIA, a group of the NFI family, was originally found to play a role in adenovirus DNA replication. Recently, it is reported that NFIs, especially NFIA and NFIB, also function in the occurrence or development of cancers [21]. Few reports showed that NFIA was targeted by miRNAs, such as miR-29a [22] and miR-223 [23]. In the research, we predicted and testified the association of miR-223-3p with NFIA in AC16 cells. Silencing of NFIA partly rescued the suppression of miR-223-3p inhibitor on cell apoptosis and autophagy in AC16 cells treated with an anthracycline, suggesting that between miR-223-3p and NFIA existed molecular regulation way in AC16 cells. At the bottom of this study, we performed rescue experiments to further inquire about the regulation mechanism of apoptosis and autophagy between NFIA and miR-223-3p. It was improved that the down-regulation of the NFIA party reversed the protection of miR-223-3p inhibitor in anthracycline-treated AC16. All data manifested that miR-223-3p dampened the apoptosis and autophagy via negatively regulating NFIA expression, which was a new molecular regulation mechanism in the process of anthracycline-induced cardiomyocyte damage.

In conclusion, all findings demonstrated that the knockdown of miR-223-3p protected AC16 cells by inhibiting cell apoptosis and autophagy. MiR-223-3p inhibitor exerted protective functions by targeting NFIA. Our study demonstrated the regulation mechanism and function between NFIA and miR-223-3p in AC16 cells *in vitro*. The function and molecular regulation of miR-223-3p and NFIA need to be confirmed *in vivo* in the future. Furthermore, this study provided a novel and promising therapeutic target for anthracycline-induced cardiomyocyte damage.
